# A curriculum to teach medical students to care for people with disabilities: development and initial implementation

**DOI:** 10.1186/1472-6920-9-78

**Published:** 2009-12-30

**Authors:** Andrew B Symons, Denise McGuigan, Elie A Akl

**Affiliations:** 1 Department of Family Medicine, University at Buffalo School of Medicine and Biomedical Sciences, Buffalo, New York, USA; 2 Department of Medicine and Department of Family Medicine, University at Buffalo School of Medicine and Biomedical Sciences, Buffalo, New York, USA

## Abstract

**Background:**

Lack of knowledge and skills, and negative attitudes towards patients with disabilities, may adversely affect the services available to this group and negatively affect their health outcomes. The objective of this paper is to describe the development and initial implementation of a curriculum for teaching medical students to care for patients with disabilities.

**Methods:**

We followed the six-step approach for developing curricula for medical education: general needs assessment, specific needs assessment, defining goals and objectives, determining the educational strategies, planning the implementation, and developing an evaluation plan.

**Results:**

The curriculum has well defined goals and objectives covering knowledge, attitudes and skills. It employs both traditional and non-traditional teaching strategies. The implementation is planned over the four-year medical school curriculum in collaboration with a number of academic departments and specialized community-based agencies. The curriculum evaluation includes an attitudinal survey which is administered using a controlled design (pre- and post- exposure to the curriculum). The initial implementation of the curriculum has been very successful.

**Conclusion:**

We have developed a longitudinal curriculum to teach medical students to care for people with disabilities. A rigorous evaluation of the impact of the curriculum is needed.

## Background

The Americans with Disabilities Act of 1990 defines "disability" as physical or mental impairment that substantially limits one or more of the major life activities of the individual[1]. Healthy People 2010 identifies people with disabilities as persons having an activity limitation, who use assistance, or who perceive themselves as having a disability[2]. It is estimated that at least one in eight Americans is living with a disability (34 million people total)[3]. As people live longer with chronic conditions, and the "baby boom" generation approaches later life, the number of people with disability or at risk for a disability is projected to increase[4,5].

The Surgeon General acknowledged in his 2005 "Call to Action" on the 15th anniversary of the Americans with Disabilities Act that, for too long, we provided lesser care to people with disabilities[4]. Indeed, most physicians focus on the restriction, isolation, and dysfunction of patients with disabilities, seeing this as the defining characteristic of the patient[6,7]. Many patients with disabilities find their doctors insensitive or patronizing, seeing them as poor, suffering victims in need of pity[8]. Patients with disability report that at visits for issues not related to their disability, their physician still insists on focusing on the disability[9,10]. Though often subtle, and rarely overtly hostile, negative provider attitudes and focusing on the disability rather than on the person can result in withholding treatment, giving inferior treatment, and neglecting general and preventive care[6,11]. For instance, assumptions that a physically disabled person is also cognitively disabled may lead to not believing a patient when he/she reports a symptom or element of medical history. Likewise, a doctor may downplay the importance of a pelvic exam in a patient with a spinal cord injury, assuming she is not sexually active. Perception that the quality of life of a disabled patient is "poor anyway" may lead to less aggressive treatment of acute problems[5,9]. Negative attitudes about patients with disabilities may compound adverse outcomes and limit services available to people with disabilities[7].

According to a recent report of the Institute of Medicine titled "Future of Disability in America," barriers to receiving health care are not only physical (e.g., inaccessibility of facilities and equipment to people with disabilities), but they are also and perhaps more importantly related to the knowledge and attitudes of health care providers[12]. In fact, people with disabilities have cited negative attitudes and behaviors of health care providers as the most formidable barriers to accessing health care services[2,6,7,9,13]. Also, medical students, residents, and practicing physicians have demonstrated deficiencies in working knowledge of even the most common forms of disability, such as cerebral palsy and learning disabilities[6,9,12,14,15].

Health care providers appear to lack the necessary education and training to care for patients with disabilities. Medical students are often uncomfortable interacting with patients with disabilities[14,16]. A survey of senior pediatric residents demonstrated a marked lack of training and confidence in prescribing therapies and devices to children with special needs[15]. A large majority of practicing physicians in California acknowledged a need for training in disabilities, with only 22% reporting any training at the medical school level[17]. Patients with disabilities often report needing to educate their physicians about basic elements of their disability[18,19]. Similarly, staff at doctors' offices are often not familiar with the special needs of patients with disabilities, and this has been linked back to the lack of awareness of the physician[16].

There is evidence that certain educational interventions such as early and frequent encounters with people with disabilities improve medical students' knowledge, attitudes and skills necessary for caring for these people[6,7,17,20,21]. However, little attention has been devoted to the development of curricular content and strategies to prepare students to deal with patients with disabilities. The objective of this paper is to describe the development and initial implementation of a curriculum for teaching medical students to care for patients with disabilities.

## Methods

We followed the six-step approach for developing curricula for medical education designed by Kern, Thomas, Howard and Bass: (1) problem identification and general needs assessment; (2) needs assessment of targeted learners; (3) goals and objectives; (4) educational strategies; (5) implementation; and (6) evaluation and feedback[22].

First, we conducted a general needs assessment through a review of the published literature and reports by national and international agencies on the need for improving both care and teaching relating to disabilities.

Second, we conducted a specific needs assessment with medical students and medical educators at the State University of New York (SUNY) at Buffalo. The needs assessment consisted of formal and informal discussions with third-year medical students during their Family Medicine clerkship rotation and with Family Medicine residents. We also held formal discussions with the directors of the introduction to clinical medicine course (years one and two of medical school), with directors of clerkships (years three and four of medical school) and with the residency program directors of Family Medicine, Internal Medicine, Pediatrics, and Physical Medicine and Rehabilitation. The discussions addressed the faculty's perceived need for, and willingness to integrate, our proposed curriculum into their rotations and/or courses.

We also conducted a specific needs assessment with community-level stakeholders. We thus established contact with the three major community-based agencies specializing in health and social services for people with disabilities (People Inc. http://www.people-inc.org, Aspire of Western New York http://www.aspirewny.org, and the Western New York Independent Living Project, Inc. http://wnyilp.org). We subsequently held formal meetings and discussions with the agencies' administrators, physicians, nurses, social workers and staff. We also held similar discussions with people with disabilities and family members of people with disabilities.

Third, we used the results of the general and specific needs assessments to develop the goals and objectives of the curriculum. For each broadly defined goal, a number of specific and measurable objectives were determined. We involved both faculty and staff from the community-based agencies in this process. We refined the goals and objectives through an iterative process of drafting, discussion and revision.

Fourth, we developed a list of educational strategies to achieve the goals and objectives of the curriculum. We scheduled the educational strategies across the four years of medical school in order to achieve a continuous and sequentially logical implementation of the curriculum. We again involved both faculty and staff from the community-based agencies in this process.

Fifth, we planned the integration of the curriculum into existing course curricula at different levels. At the medical school level, we obtained the approval of the course directors and clerkship directors to integrate the curriculum into the four-year medical studies curriculum. At the community-based agencies level, we built a collaborative team of physicians, social workers, nurses, community educators, administrators, and people with disabilities along with their families and caregivers. The collaborative team was in charge of ensuring and managing the necessary human and other resources for the adequate implementation of the curriculum. We used a matrix to map out the strategies against the year of medical school. We refined the matrix through an iterative process of drafting, discussion and revision. We also applied for federal funding of this project.

Sixth, we developed an evaluation plan for the curriculum. The plan included the type of study design, the measurement instruments, the data collection process, the data analysis plan and the dissemination plan.

## Results

### Problem identification and general needs assessment

Our literature review found that both international and national organizations called to strengthen education regarding care for patients with disabilities. At the national level, the Healthy People 2010 initiative of the U.S. Department of Health and Human Services[2] recognizes people with disabilities as a vulnerable and at-risk population, subject to health care disparities and deserving of equal access to comprehensive, culturally competent, community-based health care. The initiative calls for challenging health provider misconceptions that pose barriers to quality care, as well as for developing model curricula in areas such as health care communication. The Association of American Medical Colleges has recommended that medical schools assess their existing curricula for teaching culturally competent care and health care disparities[23]. On the international level, the World Health Organization (WHO) Disability and Rehabilitation Action Plan 2006-2011[24] proposes the development of a multi-country action-learning and research initiative to create a new paradigm of medical care for disabled persons. In describing this plan, WHO recognizes that rehabilitation is rarely included in the curriculum of medical schools. It proposes to create a curriculum on disability for medical schools as well as other health care institutions.

### Specific needs assessment

Our local specific needs assessment with both stakeholders and targeted learners found a general agreement that little attention is currently devoted to educating medical students about disability and exposing students to people with disabilities. Fourth-year medical students in a family medicine sub-internship confirmed that care for patients with disabilities had not been addressed specifically during their medical school experience. Although residents in our system provide care to patients with disabilities, they did not feel that medical school prepared them to do so. Faculty indicated there was a lack of attention in the current curriculum to issues of caring for patients with disabilities. The medical school leadership expressed a need and a willingness to incorporate modules addressing this topic. Stakeholder representatives confirmed the need to strengthen medical student exposure and education in this area, and agreed to partner with us and support our efforts to develop and implement a model curriculum to enhance medical education.

### Goals and objectives

We defined three general goals for the curriculum relating to (1) building the required knowledge, (2) instilling the appropriate attitudes, and (3) fostering the needed skills to care for people with disabilities. Table 1 lists these general goals with their respective specific objectives.

**Table 1:** The general goals and specific objectives of the curriculum to teach medical students to care for people with disabilities.

Goals	Specific objectives
**Goal 1**: To build general knowledge of common disabilities, and to dispel misconceptions and misunderstandings	Students will acquire knowledge about patient-centered care for patients with disabilities including:
	• the types, nature, frequency and causes of common disabilities;
	• the common health and behavioral problems in people with disabilities;
	• the impact of a disability on the individual and his/her family;
	• the available community resources, services, and medical and non-medical referrals;
	• the principles and clinical approaches to meeting the needs of people with disabilities.

**Goal 2**: To instill altruistic attitudes and commitment to patient-centered care for people with disabilities.	Students will demonstrate attitudes which promote patient-centered care for patients with disabilities including:
	• looking beyond the disability and seeing the individual;
	• respecting and appreciating the rights and wishes of people with a disabilities;
	• being open to examining one's own attitudes about disability;
	• respecting care givers' and families' input and needs.

**Goal 3**: To foster skills necessary for patient-centered care for people with disabilities.	Students will demonstrate skills for caring for patients with disabilities including:
	• effective communication with people with disabilities and with their families;
	• effective physical examination, assessment and
	• diagnosis of people with disabilities;
	• appropriate handling of "patient consent" prior to invasive procedures;
	• appropriate referral to and ability to access and interact with community organizations and specialists.

### Educational strategies

The educational strategies include both traditional teaching strategies such as didactic sessions and less traditional strategies such as encounters with families of patients with disabilities:

• **School-based education**: consists of didactic teaching and encounters with standardized patients with disabilities.

• **Community-based experiences**: include encounters with patients with disabilities, meetings with families of patients with disabilities, presentations by patient advocates, and visits to the specialized community agencies serving people with disabilities.

• **Clinical experiences**: consist of precepted clinical experiences in local clinics which provide primary care and integrated services for patients with disabilities.

• **Research experiences**: consist of mentored research opportunities relating to people with disabilities. One particular area of interest is the provision of community-based primary care services for people with disabilities.

### Implementation of the curriculum

Table 2 presents the matrix of a longitudinal implementation of educational strategies by year of medical school, as follows:

**Table 2:** The matrix of educational strategies by year of the curriculum to teach medical students to care for people with disabilities.

	1^st ^year	2^nd ^year	3^rd ^year	4^th ^year
Didactic teaching	*	*	*	*

Standardized patients		*		

Encounters with patients	*	*	*	*

Meetings with families	*			*

Presentations by patient advocates	*		*	*

Visits to community organizations			*	*

Clinical exposure	*		*	*

Research opportunity	*	*	*	*

• First year: a lecture presentation about the history of disabilities and society followed by small seminar group encounters with patients with disabilities and their families are integrated in the Introduction to Clinical Medicine course. The topic of discussion in the small seminar groups is "things that have been helpful and hurtful in our interactions with the health care system." Students also have presentations by patient advocates. Students may also participate in a Family Medicine summer research internship focusing on topics related to provision of health care for people with disabilities.

• Second year: a lecture presentation on clinical skills for interviewing and examining patients with disabilities is integrated in the second-year Introduction to Clinical Medicine course. Following the presentation, the students participate in an objective standardized clinical encounter (OSCE) in which selected people with disabilities are trained as standardized patients. Both the lecture and the OSCE activity are developed with the Department of Physical Medicine and Rehabilitation.

• Third year: curriculum activities are integrated within the Family Medicine and Internal Medicine Clerkship rotations. During the Family Medicine rotation, the students participate in a half-day seminar on the social context of caring for patients with disabilities. Topics include models for addressing disability in society, economic and social organization of care for people with disability, and issues of consent and guardianship. This seminar is in cooperation with a local organization which provides social and medical services for people with disabilities. During this rotation, students spend one day in a precepted clinical experience in a facility which provides primary care for patients with disabilities. During the Internal Medicine rotation, students experience a didactic presentation on common medical concerns of patients with disabilities.

• Fourth year: students may choose to participate in a four-week elective in primary care for patients with disabilities. This elective includes a variety of experiences including clinical encounters with patients, meetings with their families, meetings with patient advocates, and participation in the activities of specialized community-based agencies. Students may also enroll in a four-week mentored research elective.

### Evaluation and feedback

Our curriculum evaluation includes an attitudinal survey which is administered using a controlled design (pre- and post- exposure to the curriculum). The intervention group is a class of medical students at our university while the control group is a class of medical students at another medical school in our state. We administered the attitudinal survey to both groups before the start of the curriculum, and we will administer it a second time after the completion of the curriculum. We have developed this attitudinal survey instrument for specifically measuring attitudes about people with disabilities. The development process started with an extensive review of the literature to identify existing instruments. We then adapted these instruments guided by input from local professionals with expertise in disabilities. The tool is currently being validated.

In addition to the attitudinal survey, we are assessing individual elements of the curriculum. We are asking students to complete a reflective piece following the small seminar group encounters with patients with disabilities and their families and following their third-year precepted clinical experience. The following are excerpts from the reflective pieces completed by students following the small seminar group encounters with patients with disabilities and their families:

• "Interaction of students with real life patients early on in their medical education helps eliminate many stereotypes and prejudices...This will benefit students when they come across patients in their medical career."

• "We met a young woman with cerebral palsy...I felt myself quiet down with my questions. I didn't want to offend her by questioning her abilities...we relaxed and I was able to address her directly...this provided a valid time for introspection on our own beliefs of how we react to a particular situation."

• "...don't assume anything about people with disabilities when you initially meet them."

• "I was particularly struck by Dr. X., the physician who sustained a traumatic brain injury as a medical student...when he entered our room, I immediately jumped to certain conclusions...what a perfect example of the ease with which we make assumptions unless we train ourselves not to."

• "This was the first step in opening our eyes to the necessity of being able to fully understand what it means to care for those that may have an impaired ability to care for oneself."

• "Getting the opportunity to talk to a disabled patient and hear her voice her concerns regarding how doctors treat her made me realize how important it is to treat them the same as you would treat any other patient."

We also use the OSCE in our evaluation process. Students participating in the research electives receive summative and formative feedback from their research mentors. Students participating in the fourth-year elective receive multi-source ("360-degree") evaluation by their clinical preceptors, the support staff, their peers, and patients.

## Discussion

We have developed a longitudinal curriculum to teach medical students to care for people with disabilities using the six-step approach of Kern, Thomas, Howard and Bass for curriculum development[22]. The curriculum has well defined goals and objectives and employs both traditional and non-traditional teaching strategies. The implementation is planned over the four-year medical school curriculum in collaboration with a number of academic departments and specialized community-based agencies. The curriculum evaluation includes an attitudinal survey which is administered using a controlled design and is administered to participants before beginning the curriculum as well as after their participation in the curriculum.

Our curriculum received federal funding from the U.S. Department of Health and Human Services, Health Resources and Services Administration in June 2008 and is currently being implemented. Although the current first-year class will be the only class to experience the entire curriculum, we have been implementing elements of this curriculum with the current third-year class as well. Students currently in the second year of medical school will experience the activities in their third and fourth years. The second-year OSCE activity is still in its planning stages, which is why the second-year class has not participated in this activity.

Introducing new material into the medical school curriculum needs to be done with sensitivity to the perception that a new "special interest" will supplant existing elements, and excessively burden those responsible for delivering the existing curriculum. For that purpose we have secured a "buy-in" during the needs-assessment portion of the project. In the implementation phase, we are taking care not to overburden any one course or rotation with too many activities related to our curriculum. Faculty members have been very receptive and cooperative in incorporating elements of the curriculum.

Students in the first-year clinical skills course participated in the lecture presentation about the history of disabilities and society followed by small seminar group encounters with patients with disabilities and their families. The session was very informative and personal, providing an opportunity for the students to meet real-life patients with real-life struggles. As a result of the session, the students were able to examine and reflect on their own attitudes about disability. In the third year, students participating in the Internal Medicine Clerkship have been participating in the one-hour presentation on common medical concerns of patients with disabilities.

Students in the Family Medicine Clerkship have been attending a half-day seminar on the social context of caring for patients with disabilities and have been spending one day in a precepted clinical experience in a facility which provides primary care for patients with disabilities. Student reflections on their experiences have been informative. While students noted many similarities with their other clinical experiences, they also noted a number of unique aspects. For example, in relation to the specialized community-based facility, students commented that: the facility was more comprehensive and better run than other clinics; every patient had an accompanying advocate who used detailed records for the patient, making the physician's history taking easier; physical examinations were tailored to meet the individual needs; and more time was allotted for each patient. Most students indicated they would likely treat patients with disabilities in the future, having spent time in this clinic. One student stated, "before this experience, I had reservations about disabled patients, but I am now more comfortable with working with them in the future."

We believe that the major strength of this curriculum is the introduction of students to caring for patients with disabilities early in their career. By integrating the elements of the curriculum into other primary care-oriented courses and clerkships, students perceive caring for patients with disabilities as a natural part of patient care in general. Also, we feel that the lessons and experience gained in caring for patients with a particular disability will be transferable not only to patients with various disabilities, but also to fostering professionalism in the compassionate, competent care of all patients.

A highly gratifying part of developing and implementing the curriculum has been the partnerships fostered with the specialized community-based agencies caring for patients with disabilities, People Inc. and Aspire of Western New York. There is a great mutual appreciation in that we have given them access to medical students so as to instill in these young physicians the knowledge, attitudes, and skills necessary to care for their patients who have disabilities. In turn, we have appreciated their expertise and their contribution to the development and implementation of the curriculum.

The challenges involved in this curriculum are mostly related to its implementation in the field. Full implementation involves coordinating clinical experiences in the community, as well as training people from the community to come in and work with the students. Each of the agencies and groups with whom we interact has its own agenda and its own logistical and financial constraints. Clarifying these agendas and working within these constraints is essential for successful implementation of the program.

## Conclusion

We hope that the longitudinal curriculum to teach medical students to care for people with disabilities will inspire other educational institutions to implement similar educational initiatives. We are willing to make elements of our curriculum available to the community of medical educators working in this area. We also aim to advance the field by developing and validating outcome measurement tools and subsequently conducting experimental work to evaluate the real impact of our curriculum.

## Competing interests

The authors declare that they have no competing interests.

## Authors' contributions

ABS: substantial contributions to research design, the acquisition, analysis and interpretation of data; drafting the paper; approval of the submitted and final versions. DM: substantial contributions to the interpretation of data; revising the paper critically; approval of the submitted and final versions. EAA: study design, data analysis and interpretation, revising the paper critically; approval of the submitted and final versions.

## Pre-publication history

The pre-publication history for this paper can be accessed here:

http://www.biomedcentral.com/1472-6920/9/78/prepub

## References

[B1] The Americans with Disability Act http://www.ada.gov/pubs/ada.htm

[B2] U.S. Department of Health and Human Services Healthy people 2010 2000 Washington, DC: U.S. Dept. of Health and Human Services

[B3] Fast Stats A to Z: Disabilities or Limitations http://www.cdc.gov/nchs/fastats/disable.htm

[B4] The Surgeon General's call to action to improve the health and wellness of persons with disabilities http://www.surgeongeneral.gov/library/disabilities/20669510

[B5] Iezzoni LI Going beyond disease to address disability New England Journal of Medicine 2006 355 10 976 979 10.1056/NEJMp068093 16957143

[B6] Jackson KB Knowledge and attitudes toward persons with physical disabilities of healthcare trainees Master Thesis 2007 Roosevelt University, Clinical Psychology Department

[B7] Tervo RC Palmer G Redinius P Health professional student attitudes towards people with disability Clinical Rehabilitation 2004 18 8 908 915 10.1191/0269215504cr820oa 15609846

[B8] Byron M Cockshott Z Brownett H Ramkalawan T What does "disability" mean for medical students? An exploration of the words medical students associate with the term "disability" Medical Education 2005 39 2 176 183 10.1111/j.1365-2929.2004.02062.x 15679685

[B9] Drainoni M Lee-Hood E Tobias C Bachman S Andrew J Maisels L Cross-disability experiences of barriers to health-care access Journal of Disability Policy Studies 2006 17 101 115 10.1177/10442073060170020101

[B10] Branigan M Stewart DE Tardif GS Veltman A Perceptions of primary healthcare services among persons with physical disabilities - part 2: quality issues MedGenMed 2001 3 2 19 11549968

[B11] Paris MJ Attitudes of medical students and health-care professionals toward people with disabilities Archives of Physical Medicine & Rehabilitation 1993 74 8 818 825 10.1016/0003-9993(93)90007-W 8347067

[B12] Field MJ Jette AM Institute of Medicine (U.S.), Committee on Disability in America: a New Look The Future of Disability in America 2007 Washington, DC: National Academies Press 20669428

[B13] Byron M Dieppe P Educating health professionals about disability: 'attitudes, attitudes, attitudes' Journal of the Royal Society of Medicine 2000 93 8 397 398 10983497 10.1177/014107680009300801 PMC1298077

[B14] Martin HL Rowell MM Reid SM Marks MK Reddihough DS Cerebral palsy: what do medical students know and believe? Journal of Paediatrics & Child Health 2005 41 1-2 43 47 10.1111/j.1440-1754.2005.00534.x 15670223

[B15] Sneed RC May WL Stencel CS Training of pediatricians in care of physical disabilities in children with special health needs: results of a two-state survey of practicing pediatricians and national resident training programs Pediatrics 2000 105 3 Pt 1 554 561 10.1542/peds.105.3.554 10699109

[B16] Jain S Care of patients with disabilities: an important and often ignored aspect of family medicine teaching Family Medicine 2006 38 1 13 15 16378252

[B17] Larson McNeal MA Carrothers LA Premo B Providing primary health care for people with physical disabilities: a survey of California physicians 2002 Center for Disability Issues and the Health Professions

[B18] Kroll T Beatty PW Bingham S Primary care satisfaction among adults with physical disabilities: the role of patient-provider communication Managed Care Quarterly 2003 11 1 11 19 12790061

[B19] Morrison EH George V Mosqueda L Primary care for adults with physical disabilities: perceptions from consumer and provider focus groups Family Medicine 2008 40 9 645 651 18830840

[B20] Kahtan S Inman C Haines A Holland P Teaching disability and rehabilitation to medical students. Steering Group on Medical Education and Disability Medical Education 1994 28 5 386 393 10.1111/j.1365-2923.1994.tb02549.x 7845257

[B21] Thistlethwaite JE Ewart BR Valuing diversity: helping medical students explore their attitudes and beliefs Medical Teacher 2003 25 3 277 281 10.1080/0142159031000100346 12881050

[B22] Kern DE Thomas PA Howard DM Bass EB Curriculum Development for Medical Education: A Six-Step Approach 1998 Baltimore: Johns Hopkins University Press

[B23] Tool for Assessing Cultural Competence Training (TACCT) http://www.aamc.org/meded/tacct/start.htm

[B24] Disability and Rehabilitation WHO Action Plan 2006-2011 http://www.who.int/disabilities/publications/dar_action_plan_2006to2011.pdf

